# Axitinib induces senescence-associated cell death and necrosis in glioma cell lines: The proteasome inhibitor, bortezomib, potentiates axitinib-induced cytotoxicity in a p21(Waf/Cip1) dependent manner

**DOI:** 10.18632/oncotarget.13769

**Published:** 2016-12-01

**Authors:** Maria Beatrice Morelli, Consuelo Amantini, Massimo Nabissi, Claudio Cardinali, Matteo Santoni, Giovanni Bernardini, Angela Santoni, Giorgio Santoni

**Affiliations:** ^1^ School of Pharmacy, University of Camerino, Camerino, Italy; ^2^ Department of Molecular Medicine, Sapienza University, Rome, Italy; ^3^ School of Biosciences and Veterinary Medicine, University of Camerino, Camerino, Italy; ^4^ Department of Medical Oncology, Polytechnic University of Marche, Ancona, Italy; ^5^ I.N.M. Neuromed, Pozzilli, Isernia, Italy

**Keywords:** axitinib, glioblastoma, bortezomib, p21, senescence

## Abstract

Glioblastoma is associated with a poor overall survival despite new treatment advances. Antiangiogenic strategies targeting VEGF based on tyrosine kinase inhibitors (TKIs) are currently undergoing extensive research for the treatment of glioma.

Herein we demonstrated that the TKI axitinib induces DNA damage response (DDR) characterized by γ-H2AX phosphorylation and Chk1 kinase activation leading to G2/M cell cycle arrest and mitotic catastrophe in U87, T98 and U251 glioma cell lines. Moreover, we found that p21(Waf1/Cip1) increased levels correlates with induction of ROS and senescence-associated cell death in U87 and T98 cell lines, which are reverted by N-acetyl cysteine pretreatment. Conversely, U251 cell line showed a resistant phenotype in response to axitinib treatment, as evidenced by cell cycle arrest but no sign of cell death.

The combinatorial use of axitinib with other therapies, with the aim of inhibiting multiple signaling pathways involved in tumor growth, can increase the efficiency of this TKI. Thus, we addressed the combined effects of axitinib with no toxic doses of the proteasome inhibitor bortezomib on the growth of U87 and T98 axitinib-sensitive and axitinib-resistant U251 cell lines. Compared to single treatments, combined exposure was more effective in inhibiting cell viability of all glioma cell lines, although with different cell death modalities. The regulation of key DDR and cell cycle proteins, including Chk1, γ-H2AX and p21(Waf1/Cip1) was also studied in glioma cell lines.

Collectively, these findings provide new perspectives for the use of axitinib in combination with Bortezomib to overcome the therapy resistance in gliomas.

## INTRODUCTION

Glioblastoma (GBM) is a high angiogenic malignancy. GBM secretes high levels of vascular endothelial growth factor (VEGF) that promotes endothelial cell proliferation, blood brain barrier permeability and angiogenesis [1, 2]. They are aggressive tumors that generally respond poorly to therapy consisting of surgery, radiation, and conventional chemotherapy. Although advancements in the past decades, no significant increase in the overall survival (OS) of patients is observed, with a median survival of 14.6 months and five-year survival < 10% [3]. Molecularly targeted agents hold significant promise as novel therapeutic adjuncts; however, these new therapies are still in clinical trial phase. Among targeted therapies, a new current focuses on the angiogenic tyrosine kinase receptors (TRKs) and their signaling pathway inactivation. The anti-VEGF antibody bevacizumab was the first TKI approved in 2009 by the Food and Drug Administration (FDA) as a second-line treatment of recurrent GBM [4].

A novel orally available TKI is axitinib (Inlyta®), a selective and potent inhibitor of VEGFR 1, 2, and 3 that has been approved by FDA in 2012 for the treatment of patients with metastatic renal cell carcinoma (mRCC) after failure of one prior systemic therapy [5, 6]. We have recently reported that axitinib induces activation of DNA damage response (DDR), senescence and mitotic catastrophe in RCC cell lines [7]; however at present, very few data using this TKI have been provided in GBM. Preclinical study showed that systemic treatment with axitinib exerts antiangiogenic effect and survival prolongation in preclinical orthotopic GBM models, including clinically relevant glioma stem cell models [8]. More recently, a randomized multicenter phase II clinical trial demonstrated an increased 6-month progression-free survival (PFS) rate of 34% after axitinib treatment in patients with recurrent GBM with respect to 28 % PFS in the control patients treated with bevacizumab or lomustine, suggesting that in recurrent GBM, axitinib shows clinical activity as single-agent [9]. Finally, a window study on front-line axitinib followed by axitinib and radiation in elderly patients with glioblastoma from University of Cincinnati (NCT01508117) is currently under evaluation.

Other new-targeted therapies interfere with proteasome, the enzymatic complex responsible for the bulk of protein degradation. Proteasome inhibition leads to toxic accumulation of misfolded and abnormal proteins and can also stabilize specific tumor inhibitory factors such as cell cycle regulatory proteins and induce apoptosis [10–14]. The proteasome inhibitor bortezomib (PS-341/Velcade) is FDA-approved for multiple myeloma and mantle cell lymphoma [15]. Bortezomib functions as a selective inhibitor of the 26S proteasome, producing predictable, dose-related, and reversible proteasome inhibition [16]. It exerts antitumor activity in a variety of malignancies [17]. *In vitro* studies have demonstrated that bortezomib alone or in combination with histone deacetylase (HDAC) [18], the cyclooxygenase-2 inhibitor celecoxib (Celebrex) [19], phosphatidylinositol 3-kinase (ZSTK474) inhibitors [20] or temozolomide [21, 22] stimulates a potent cytotoxic response and causes cell death in GBM cell lines.

Therefore, the aim of the present work was to evaluate the effects of axitinib treatment as monotherapy and in combination with bortezomib on multiple signaling pathways involved in glioma growth. Of particular interest was the cytotoxic synergy of axitinib-bortezomib combination found in different human glioma cell lines that involves the modulation of p21 (Waf1/Cip1) protein levels and leads to enhanced cell death.

## RESULTS

### Axitinib inhibits glioma cell viability in a dose and time-dependent manner

**Figure 1:**
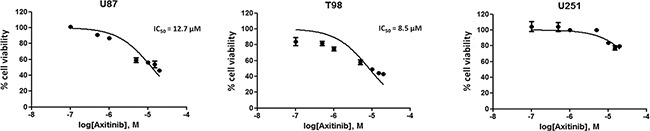
Axitinib inhibits viability in glioma cell lines U87, T98 and U251 glioma cell lines were cultured for 72 h with different doses of axitinib. Cell viability was determined by MTT assay. Data shown are expressed as mean ± SE of three separate experiments.

We first evaluated the effects of axitinib on cell viability in U87, T98 and U251 glioma cell lines by performing dose-response and time-course analyses (Supplementary Figure S1A). Axitinib inhibited the growth of U87 and T98 cells, after 72 h of treatment, with IC_50_ values of 12.7 µM and 8.5 µM, respectively (Figure 1). Conversely, U251 cells were found to be more resistant to axitinib-mediated cytotoxic effects. Therefore, the lowest effective dose of axitinib in inducing growth inhibition for each cell line (5 μM for U87 and T98; 15 μM for U251) was used for the subsequent experiments.

### Axitinib triggers the DNA damage response (DDR) and p21 overexpression in glioma cell lines

**Figure 2:**
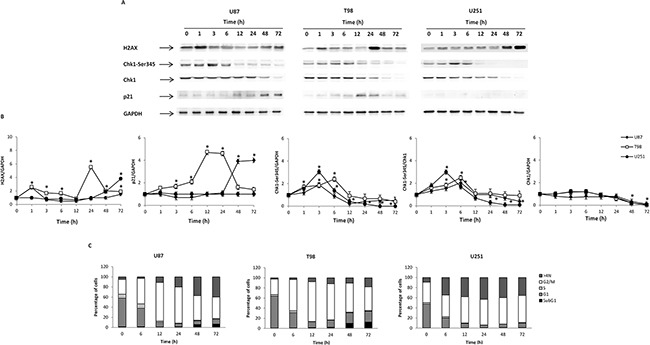
Axitinib induces DNA damage response and cell cycle arrest **A**. Western blot analysis of H2AX, Chk1-Ser345, Chk1 and p21 protein levels in glioma cells after 72 h treatment with 5 μM axitinib for U87 and T98 cells, and with 15 μM axitinib for U251. Blots are representative of one of three separate experiments. **B**. H2AX, Chk1-Ser345, Chk1 and p21 densitometry values were normalized to GAPDH used as loading control. The Chk1-Ser345 protein levels were also determined with respect to Chk1 levels. Densitometric values shown are the mean ± SD of three separate experiments. *p<0.01 *vs* untreated cells. **C**. Cell cycle analysis in U87 and T98 cells treated with 5 μM axitinib and in U251 cells treated with 15 μM axitinib for the indicated times.

Axitinib has been found to trigger DDR in RCC lines [7], however at present no data on the effect of axitinib in glioma are available. Thus, to evaluate whether axitinib treatment could trigger the DDR in glioma cells, we initially investigated the presence of γ-H2AX (H2AX), Ser139 phosphorylated variant of histone 2A associated with DNA double-strand breaks [23]. Western blot analysis revealed strong induction of the DNA damage marker expression in all axitinib-treated glioma cell lines, although with different kinetics (Figure 2A and 2B). Interestingly, phospho-H2AX induction was accompanied by Ser345-Chk1 phosphorylation already at 3 h after exposure to axitinib that declined at later time points in all glioma cell lines. The Chk1 protein was expressed in all glioma cell lines until 48 h, and declined at later time points after axitinib treatment (Figure 2A and 2B). At 12 h after treatment, p21 overexpression, that paralleled the decline of Ser345-Chk1 activation, was observed in U87 and T98 cells, but not in U251 cells (Figure 2A and 2B).

### Axitinib induces G2/M arrest and mitotic catastrophe in glioma cell lines

Then we evaluated whether axitinib treatment could result in cell cycle alteration. Thus, we performed cell cycle experiments in the presence of axitinib for different times. We observed that treatment of glioma cells induced a significant early (just at 6 h) and transient decrease of G1-phase which was accompanied by a progressive increase of G2/M-phase cell population until 24 h in U87, T98 glioma cells and 72 h in U251 (Figure 2C and Supplementary Figure S1B). In addition, a decreased percentage of U87 and T98, but not U251 cells in G2/M-phase cells paralleled by an increase of subG1 phase was observed at 48-72 h after axitinib treatment (Figure 2C and Supplementary Figure S1B). Moreover, treatment with axitinib led to a significant increase in the percentage of cells with polyploidy in all glioma cell lines analyzed (cells with DNA content >4N) (Figure 2C and Supplementary Figure S1B).

**Figure 3:**
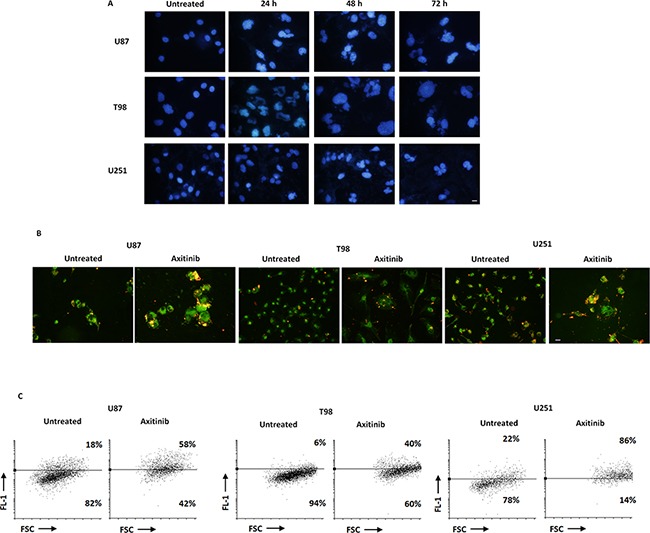
Axitinib triggers mitotic catastrophe in all glioma cell lines **A**. Nuclei of glioma cells untreated or treated with axitinib for the indicated time were stained with Hoechst 33258 and then analyzed on ten random fields. Cells were observed under a fluorescence microscope. Bar: 30 μm. **B**. Fluorescence microscope images show increase of green fluorescent signal upon axitinib treatment after 72 h compared to untreated cells. Bar: 30 μm. **C**. Change in JC1 green (FL-1) with respect to FSC parameter in cells treated with axitinib for 72 h was detected by flow cytometer.

Tetraploid tumor cells intrinsically susceptible to mitotic aberrations are particularly sensitive to the induction of mitotic catastrophe [24, 25]. Thus, we decided to investigate whether axitinib treatment could result in mitotic catastrophe in glioma cells, by assessing the changes in nuclear morphology [26]. By Hoechst 33258 staining we observed that the nuclei became significantly larger and some cells contained several nuclei of unequal sizes already after 24 h of drug treatment in U87, T98 and U251 glioma cell lines (Figure 3A). Further, in order to assess mitochondrial changes in mitotic catastrophe, JC-1 staining was used to analyze mitochondrial mass. As shown in Figure 3B and 3C, an increase of FL-1 green fluorescence in axitinib treated compared to untreated glioma cells, resulting by enhanced mitochondrial mass, was observed. This data was also confirmed by NAO staining [27] (Supplementary Figure S1C).

### Axitinib triggers senescence-associated cell death and necrosis in U87 and T98 glioma cell lines

**Figure 4:**
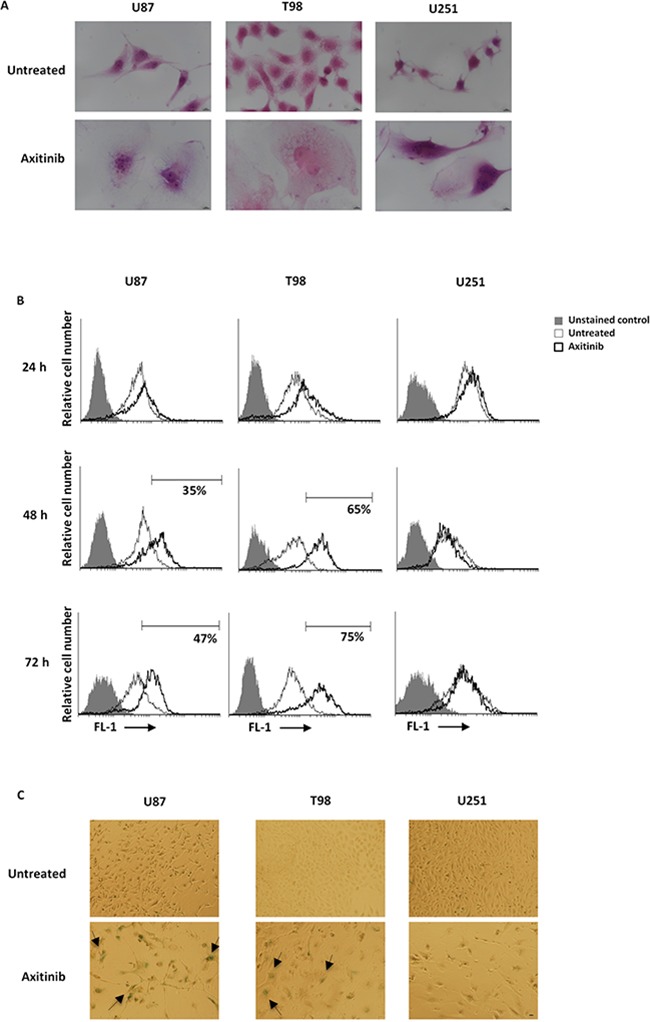
Axitinib induces cellular senescence in U87 and T98, but not in U251 glioma cell lines **A**. Representative image of glioma cells 72 h after treatment with axitinib and then stained with haematoxylin and eosin (H&E). Bar: 10 μm. **B**. Representative flow cytometric profiles of glioma cells untreated or treated with axitinib (5 μM for U87 and T98 cells and 15 μM for U251), then stained with C_12_FDG, a fluorogenic substrate for SA-β-galactosidase before analysis. Data represent the percentage of positive cells. Grey curve represents unstained cells. **C**. Cellular senescence was assessed in glioma cells after 72 h of axitinib treatment by cytochemistry detection of SA-β-galactosidase activity. Bar: 20 μm.

Cell cycle arrest in G2/M phase and polyploidy often result in cellular senescence [28]. Thus, a time-dependent accumulation of glioma cells with enlarged and flattened morphology was observed after axitinib treatment by microscopy analysis (Figure 4A). Since these morphological changes are reminiscent of a senescent phenotype, we analyzed the activity of senescence-associated β-galactosidase (SA-β-gal), the typical marker of senescent cells, in glioma cells after axitinib treatment [29, 30]. Forty-eight hours after treatment, the increase on SA-β-gal activity was detected by flow cytometry using the fluorogenic substrate for SA-β-galactosidase, C_12_FDG, and reached the percentage of 51 and 75 in U87 and T98 cells, respectively at 72 h of treatment (Figure 4B). These results were also confirmed by cytochemical assessment of SA-β-gal activity that revealed the presence of blue-stained U87 and T98 glioma cells after treatment with axitinib (Figure 4C). No SA-β-gal-positive senescent cells were found in axitinib-treated U251 glioma cells (Figure 4B, 4C).

**Figure 5:**
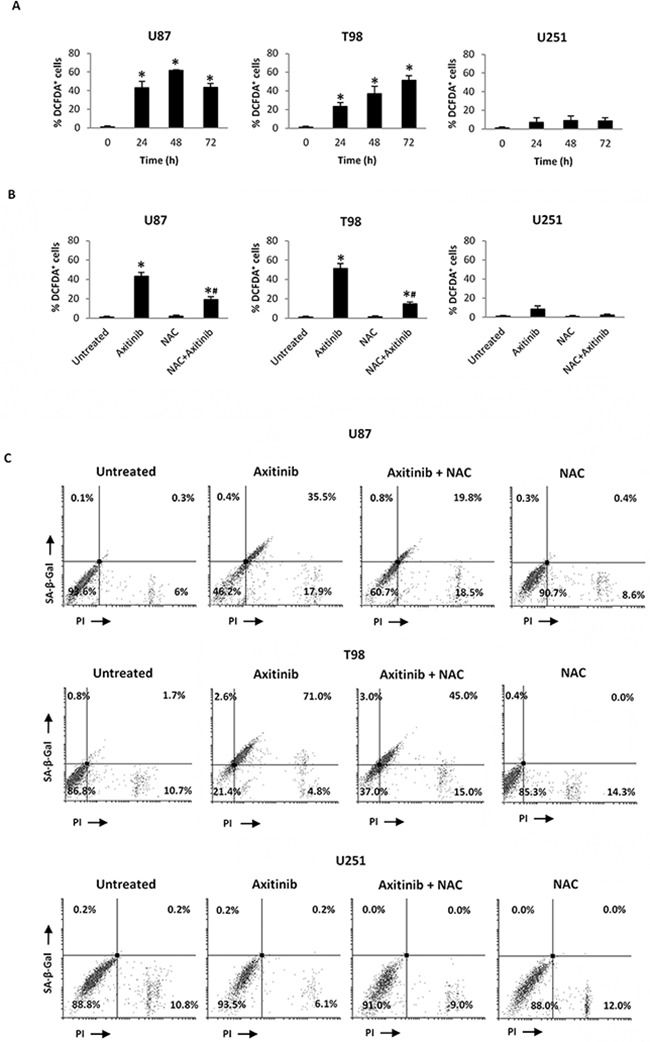
Axitinib induces ROS-dependent senescence-associated cell death in U87 and T98 cell lines **A**. ROS generation in glioma cell lines treated with axitinib (5 μM for U87 and T98 cells and 15 μM for U251) for the indicated times. Cells were stained with DCFDA before flow cytometric analysis. Data are expressed as percentage of DCFDA positive cells with respect to untreated cells; *p < 0.01 *vs* untreated cells. **B**. Glioma cells were pretreated with NAC 10 mM for 1 h before the administration of axitinib. After 72 h cells were stained with DCFDA and analyzed as above described. *p < 0.01 *vs* untreated cells; ^#^p < 0.01 *vs* axitinib or NAC treated cells. **C**. Glioma cells were cultured with axitinib for 72 h, with or without pretreatment with NAC 10 mM for 1 h. Flow cytometric analysis was performed by C_12_FDG and PI double-staining. Data represent the percentage of PI and/or SA-β-galactosidase positive cells and are representative of one of three separate experiments.

Recent studies have suggested that reactive oxygen species (ROS) generation triggered by anti-cancer drug can stimulate cellular senescence [31, 32]. Thus, we evaluated the capability of axitinib to trigger ROS generation in glioma cells by flow cytometry using the general redox-sensitive fluorescent dye, DCFDA. As shown in Figure 5A, axitinib stimulated intracellular ROS generation in U87 and T98, but not in U251 glioma cells. It was evident at 24 h after exposure and progressively increased at later time points. This response was reverted by the pre-treatment of glioma cells with the ROS scavenger, N-acetyl-l-cysteine (NAC) (Figure 5B).

Depending whether it is replicative or premature, cellular senescence may also affect cell viability [33, 34]. Thus, we evaluated the effect of axitinib exposure on glioma cell viability by SA-β-gal/PI staining and FACS analysis. An increased number of SA-β-gal^+^PI^+^ and SA-β-gal^-^PI^+^ cells, suggestive of premature senescence and necrotic cell death, respectively, was observed in U87 cells after axitinib exposure, whereas axitinib-treated T98 cells showed a SA-β-gal^+^PI^+^ phenotype (Figure 5C). Neither premature senescence or necrotic cell death were identified in axitinib-treated U251 cells (Figure 5C). Moreover, pre-treatment of axitinib-administered U87 and T98 glioma cells with NAC reduced the percentage β-gal^+^PI^+^ in axitinib-treated cells, whereas no changes in NAC-treated cells were observed (Figure 5C).

Altogether, the high percentage of SA-β-gal^+^PI^+^ U87 and T98 cells suggested that these two cell lines undergo to senescence-associated cell death, and the ROS generation is indispensable for the induction of this process. On the contrary in U251 glioma cells, the inability of axitinib to trigger ROS production results in a failure to induce premature senescence and cell death. Furthermore, the lack of Annexin V^+^ cells and caspase-3 activation up to 72 h of axitinib treatment evidenced a necrotic cell death in U87 and T98 cells (Supplementary Figure S1D, 1E).

To elucidate whether axitinib treatment was able to select resistant glioma cells, the ability to resume proliferation was evaluated. To this purpose U87, T98 and U251 cell lines were incubated with axitinib for 72 h and at the end of the treatment the viable cells remained were washed to remove axitinib, replated and incubated for additional days in fresh media. After axitinib removal, only U251 cells showed the ability to restart to growth although at lower degree respect to untreated cells (Supplementary Figure S2A). Moreover, data obtained by staining for SA-βGal/PI incorporation showed an increased percentage of SA-βGal^+^/PI^+^ and PI^+^ cells in U87 and in T98 cells, respectively. In U251 cells the absence of senescence-associated SA-βGal or PI staining respect to untreated cells after culture in fresh media was observed (Supplementary Figure S2B).

Taken together, these results demonstrated that axitinib induces an irreversible senescence and cell death in U87 and T98 cells. On the other hand, U251 cells are confirmed to show a resistant phenotype.

### Overexpression of p21 sensitizes U251 glioma cells to axitinib treatment

**Figure 6:**
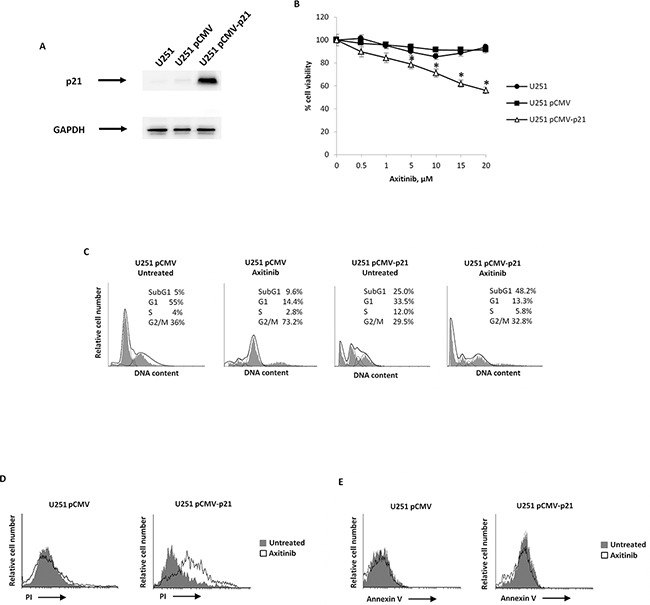
p21 overexpression decreases cell viability of axitinib-treated U251 glioma cells **A**. Lysates from untransfected, pCMV and pCMV-p21 U251 cells were separated on SDS-PAGE and probed with specific rabbit anti-human p21 Ab. GAPDH protein levels were evaluated as loading control. Blots are representative of three separate experiments. **B**. Untransfected, pCMV and pCMV-p21 U251 cell viability was determined by MTT assay after 72 h of transfection. Data shown are the mean ± SD of three separate experiments. *p<0.01 *vs* untransfected and pCMV U251 cells. **C**. Representative cell cycle distribution in pCMV and pCMV-p21 U251 cells treated for 72 h with axitinib. **D**. U251 cells were treated as above described and then PI incorporation was analyzed by flow cytometry. Histograms are representative of one of three separate experiments. **E**. U251 cells were treated as above described and then Annexin V staining was analyzed by flow cytometry. Histograms are representative of one of three separate experiments.

Owing to the potential role of p21 in the senescence pathway, we evaluated whether overexpression of exogenous p21 would sensibilize axitinib-resistant U251 cells from axitinib-induced cell death. Therefore, we transfected pCMV vector containing coding sequence for p21 in this cell line, and we investigated the cell viability. Firstly, we evaluated the p21 protein expression in pCMV (transfection control) and pCMV-p21 respect to untransfected U251 glioma cells by western blot analysis. The p21 protein levels were not detectable in untreated and pCMV U251 cells, whereas increased p21 expression was observed in pCMV-p21 U251 transfected glioma cells (Figure 6A). About 40% of growth inhibition was observed upon axitinib treatment in pCMV-p21 U251 glioma cells after 72 h of treatment (Figure 6B). In addition, p21 overexpression increased the percentage of cells in subG1 phase respect to U251 pCMV transfected cells (Figure 6C). Whereas in pCMV transfected cells axitinib treatment increased the percentage of cells in G2/M phase, in pCMV-p21 transfected cells axitinib markedly improved the subG1 phase as compared to untreated cells (Figure 6C). Accordingly, PI staining and cytofluorimetric analysis revealed, an increased percentage (about 50%) of PI^+^ cells in axitinib-treated pCMV-p21 U251 cells, as compared to untreated pCMV-p21 U251 cells (Figure 6D). However, axitinib-treated pCMV-p21 U251 cells were not Annexin V positive (Figure 6E). No major differences were found upon axitinib treatment in pCMV U251 cells.

Overall, overexpression of p21 in axitinib-resistant U251 glioma cells overcomes the U251 glioma cell dysfunction, and sensitizes glioma cells to axitinib-induced cytotoxic effects.

### Bortezomib in combination with axitinib stimulates a synergistic cytotoxic effect in glioma cell lines

Bortezomib has been found to induce p21 over expression and apoptotic cell death of glioma cell lines [35]. So, we performed a time course analysis and found that bortezomib reduces the viability in U87, T98 and U251 cells with IC_50_ values of 3.5 nM, 4.5 nM and 5.0 nM, respectively (Supplementary Figure S3). Thereafter, to evaluate the potential synergistic effects of axitinib and bortezomib, we performed MTT cell growth assays treating cells with different doses of axitinib (1, 5 and 15 μM) in combination with different doses of bortezomib (1.25, 2.5 and 6.5 nM) for up to 72 h. We evaluated the cell viability on axitinib-sensitive (U87 and T98) and axitinib-resistant U251 glioma cell lines in two different schedules. As shown in Supplementary Figure S4, the synergistic effects between axitinib and bortezomib was present only when drugs were simultaneously administered. In sequential regimens, axitinib was added for 72 h, then the viable cells were replated and treated with bortezomib for other 72 h. No improvement of cytotoxic effects was observed, rather the axitinib-pretreated cells were more resistant to bortezomib treatment (Supplementary Figure S4A). In simultaneous regimen, both drugs were added on day 0 and the combined effect was evaluated on the basis of the CI (Supplementary Figure S4B). The CI results showed synergism when axitinib and bortezomib are coadministered and the lowest dose of bortezomib displaying a CI<1 (2.5 nM) was selected to study the cytotoxic effects of simultaneous treatment. To ensure that this dose was not cytotoxic, we performed several experiments. Our results demonstrated that bortezomib used at 2.5 nM does not induce mitotic catastrophe as evaluated by nuclear morphology and mitochondrial mass (Supplementary Figure S5A-B), does not alter cell cycle phases (Supplementary Figure S5C), does not induce ROS generation (Supplementary Figure S5D) or cell senescence process (Supplementary Figure S5E), does not exert cytotoxic effects as evaluated by Annexin V-PI double staining (Supplementary Figure S5F) and does not change the VEGFA mRNA levels (Supplementary Figure S5G).

### Effects of the axitinib-bortezomib combination on cell death signaling pathways

**Figure 7:**
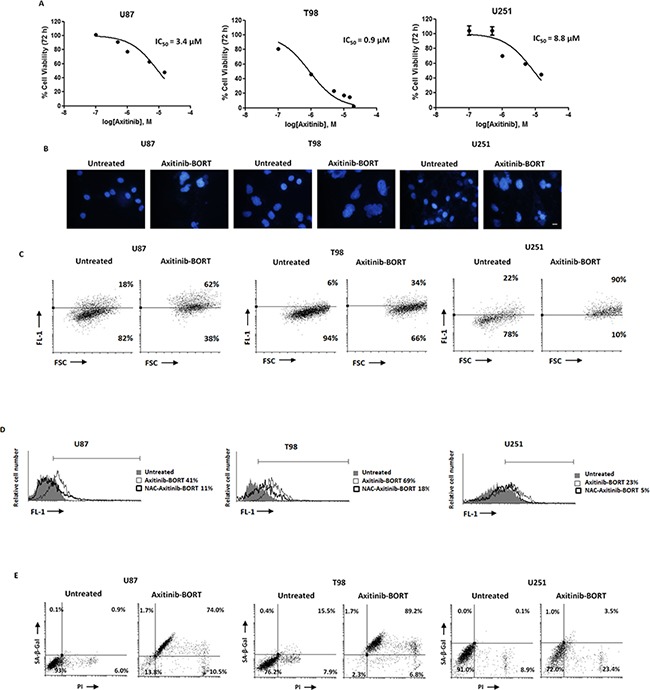
Cotreatment axitinib-bortezomib induces cell death also in axitinib-resistant U251 glioma cell line **A**. U87, T98 and U251 glioma cell lines were cultured for 72 h with different doses of axitinib administred in combination with 2.5 nM bortezomib. Cell viability was determined by MTT assay. Data shown are expressed as mean ± SE of three separate experiments. **B**. Glioma cells were cultured for 72 h with the combination axitinib (5 μM for U87 and T98, 15 μM for U251) - bortezomib (2.5 nM). Nuclei of treated cells were then stained with Hoechst 33258 and analyzed on ten random fields. Cells were observed under a fluorescence microscope. Bar: 50 μM. **C**. Change in JC1 green (FL-1) respect to FSC parameter in cells treated with the combination axitinib-bortezomib as above described was detected by flow cytometer. **D**. ROS generation in glioma cell lines cotreated for 72 h with axitinib and bortezomib, pretreated or not with NAC 10 mM for 1 h. Cells were stained with DCFDA before flow cytometric analysis. Data are expressed as percentage of DCFDA positive cells with respect to untreated cells. **E**. Flow cytometric analysis on glioma cells cultured as above described was performed by C_12_FDG and PI double-staining. Data represent the percentage of PI and/or SA-β-galactosidase positive cells and are representative of one of three separate experiments.

Axitinib plus bortezomib coadministration for 72h synergized to increase cytotoxic activity against glioma cells, as compared to axitinib alone, with a 5- and 10-fold reduction of the IC_50_ in U87 and T98 glioma cells (IC_50_ axitinib = 12.7 and 8.5 vs IC_50_ axitinib-bortezomib = 3.4 and 0.9 μM in U87 and T98 glioma cells, respectively) (Figure 7A). Furthermore, axitinib in combination with bortezomib reverted the resistance of U251 glioma cells to axitinib treatment alone and inhibited U251 glioma cell viability with an IC_50_ value of 8.8 μM (Figure 7A).

To investigate the synergistic mechanism, we evaluated the effects of bortezomib (2.5 nM) coadministered with the lowest effective dose of axitinib (5 μM in U87 and T98; 15 μM in U251).

After 72 h, all glioma cell lines underwent mitotic catastrophe, as evidenced by nuclear morphology and mitochondrial mass increase (Figure 7B, 7C and Supplementary Figure S6). Moreover, the combination of TKI and proteasome inhibitor highly increased the levels of intracellular ROS activity in U87 and T98 cell lines (Figure 7D) whereas lower ROS levels were observed in axitinib-resistant U251 glioma cells compared to cell treated with axitinib alone (Figure 7D). The ROS production effect was attenuated by NAC pretreatment after 72 h of treatment (Figure 7D). Furthermore, axitinib plus bortezomib in combination resulted in an increased percentage of β-gal^+^PI^+^ in U87 and T98 cells and β-gal^-^PI^+^ U251 cells (Figure 7E), as compared to axitinib (Figure 5C) or bortezomib (Supplementary Figure S5E, S5F) used alone. Moreover, as described for axitinib monotherapy in T98 and U87, neither Annexin V positive cells, apoptotic DNA fragmentation or caspase-3 activity (Supplementary Figure S7A-S7C) were evidenced in axitinib plus bortezomib treated U251 glioma cells. Since U251 cells resumed proliferation after axitinib removal, we investigated if the coadministration of axitinib plus bortezomib was able to induce an irreversible growth arrest.

Thus, U251 cells were treated with both drugs for 72h, then washed and replated in fresh medium. MTT results showed that the coadministration strongly inhibits the cell growth recovery suggesting that the drug combination is able to overcome U251 resistant phenotype. Bortezomib alone did not affect the rate of growth (Supplementary Figure S7D).

### Involvement of p21 in cytotoxicity induced by axitinib plus bortezomib

**Figure 8:**
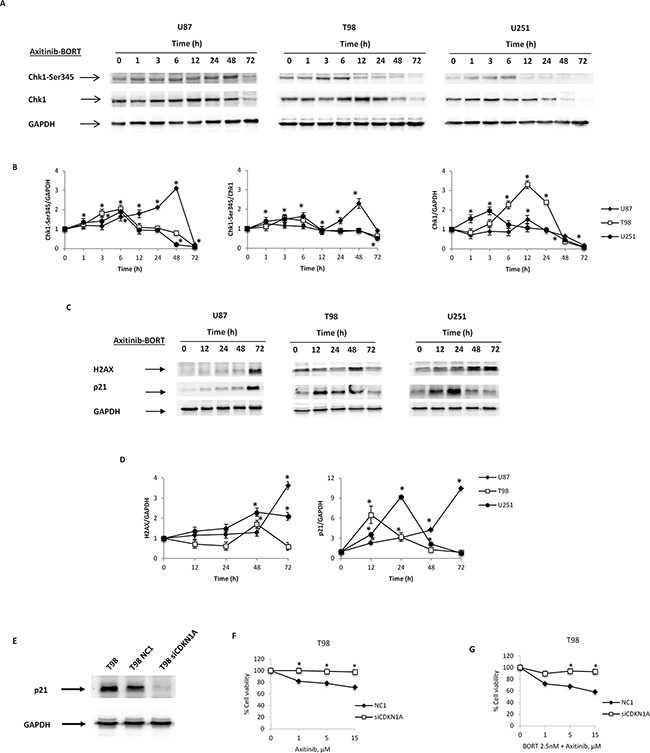
The role of p21 protein in axitinib-bortezomib induced cytotoxic effects **A**. Western blot analysis of Chk1-Ser345 and Chk1 protein levels in glioma cells after treatment with bortezomib (2.5 nM) and axitinib (5 μM for U87 and T98, 15 μM for U251). Blots are representative of one of three separate experiments. **B**. Chk1-Ser345 and Chk1 densitometry values were normalized to GAPDH used as loading control. The Chk1-Ser345 protein levels were also determined with respect to Chk1 levels. Densitometric values shown are the mean ± SD of three separate experiments. *p<0.01 *vs* untreated cells. **C**. Western blot analysis of H2AX and p21 protein levels in glioma cells after the axitinib-bortezomib combined treatment. Blots are representative of one of three separate experiments. **D**. Quantitative representation of the experiment reported in panel C. H2AX and p21 densitometry values were normalized to GAPDH used as loading control. Densitometric values shown are the mean ± SD of three separate experiments. *p<0.01 *vs* untreated cells. **E**. Lysates from untransfected, NC1 (negative control) and siCDKN1A T98 cells were separated on SDS-PAGE and probed with specific rabbit anti-human p21 Ab. GAPDH protein levels were evaluated as loading control. Blots are representative of three separate experiments. **F**. NC1 and siCDKN1A T98 cell viability was determined by MTT assay after 72 h of axitinib treatment. Data shown are the mean ± SD of three separate experiments. *p<0.01 *vs* NC1 transfected cells. **G**. NC1 and siCDKN1A T98 cell viability was determined by MTT assay after 72 h of axitinib-bortezomib cotreatment. Data shown are the mean ± SD of three separate experiments. *p<0.01 *vs* NC1 transfected cells.

The effects of axitinib plus bortezomib used in combination were associated with an enhanced p21 protein levels in all glioma cell lines with respect to cells treated with bortezomib or axitinib alone. No significative changes of Ser345-Chk1 and H2AX phosphorylation were observed (Figure 8A–8D, Supplementary Figure S8).

The fact that p21 was accumulated in all three cell lines cotreated with axitinib and bortezomib, further suggests that p21 may play an essential role in axitinib-induced cell death. Therefore, through RNA silencing (siRNA) technique we analyzed the p21 involvement in T98 cell line. After 72 h of transfection, when p21 expression is impaired by the specific siCDKN1A (Figure 8E), both axitinib- and axitinib-bortezomib-induced cytotoxicity is reduced in siCDKN1A T98 cells respect to NC1 T98 cells, used as negative transfection control (Figure 8F, 8G).

Overall, bortezomib plus axitinib in combination increase p21 levels and sensitize glioma-sensitive and resistant cells to drug-induced cytotoxic effects by triggering senescence-associated cell death and necrosis, respectively.

## DISCUSSION

In this study, we describe that axitinib treatment affects glioma cell viability inducing the DDR. This response to genotoxic insults involves sensing of DNA damage by a class of protein kinases, including ATM, ATR, followed by activation of Chk1 and Chk2 kinases that cause temporary cell cycle arrest, as well as promotes assembly of DNA repair complexes at the damaged sites at chromosomes [36]. The checkpoint regulators Chk1 and p21 were found to promote damage-induced mitotic catastrophe and a senescence-like phenotype [37].

Here, we found that treatment of glioma cells with axitinib triggers the DDR, evidenced by increased levels of phosphorylated H2AX and Ser345-Chk1, leading to cell cycle arrest at G2/M phase and accumulation of polyploid glioma cells undergoing mitotic catastrophe. Moreover, in U87 and T98 cell lines, at later time points, when Ser-345-Chk1 activation declines, the overexpression of the cell cycle inhibitor p21 activates a cell senescent program resulting in senescence-associated cell death and necrosis of treated cells. Conversely, a progressive increase of G2/M phase and an accumulation of polyploidy cells at 72 h after treatment were observed in U251 axitinib-resistant cells.

Numerous anti-tumor drugs, including DNA damaging agents, can induce mitotic catastrophe [25]. In particular, tetraploid tumor cells, intrinsically susceptible to mitotic aberrations, are sensitive to the induction of mitotic catastrophe [24, 25]. To detect the occurrence of mitotic catastrophe, both morphologic characteristics and presence of mitotic defects are used [38]. Originally, mitotic catastrophe was defined as a cell death-related process caused by aberrant mitosis. However, it has been now demonstrated that mitotic catastrophe represents a step preceding apoptosis or necrosis [24, 25]. In this regard, we found that axitinib induces mitotic catastrophe both in drug-sensitive U87 and T98 and -resistant U251 glioma cells. Indeed all glioma cell lines undergoing mitotic catastrophe show multinucleation and increased mitochondrial mass but only U87 and T98 cells die. Overall our findings support the hypothesis that mitotic catastrophe represents a step preceding a senescent-associated cell death.

At present two different types of cellular senescence have been demonstrated *in vivo* and *in vitro*. Replicative senescence represents a stable and long-term loss of proliferative capacity, despite continued viability and metabolic activity, due to telomere loss or dysfunction [33], and premature senescence, a type of DNA-damage senescent-associated cell death that arises at a stage before telomere shortening. This latter senescence program is induced by chemotherapeutic agents, oxidative stress, oncogenic or mitogenic signals [39–41, 34], and results in reduced cell viability and induction of cell death through different cell death modalities despite the p53 status of the different cell lines. Indeed we evidenced an increased number of β-gal^+^PI^+^ and β-gal^-^PI^+^ cells suggestive of senescent-associated necrosis and necrotic cell death in p53 wild-type U87 cells after axitinib exposure. In p53 mutant T98 cell line, the axitinib-treated cells showed a β-gal^+^PI^+^ phenotype, sign of a senescence-associated cell death; no senescent cell death or necrosis were evidenced in axitinib-treated p53 mutant U251 cells.

DNA damage-induced senescent necrosis is caused by an excessive intracellular ROS generation that impairs DNA repair and cell signaling [32]. In this regard, a time-dependent increase of ROS generation was observed in axitinib-sensitive U87 and T98 cells; pretreatment with the anti-oxidant agent NAC, resulted in a decreased percentage of β-gal^+^PI^+^ cells. On the contrary in U251 glioma cells, the inability of axitinib to trigger ROS signals, makes drug-treated cells resistant to premature senescence and cell death. Similarly, treatment of RCC cells and gastric cancer cells with axitinib, results in cell cycle arrest in G2 phase, and induces a ROS-dependent SA-β-galactosidase-positive senescent phenotype [7, 42].

Chk1 regulates DNA replication, cell cycle progression, chromatin remodeling and cell death. Here we found that Chk1 activation is associated with cell cycle arrest and mitotic catastrophe in all glioma cell lines tested. However, in axitinib-treated U87 and T98 cells, H2AX phosphorylation and Ser345-Chk1 activation transiently arrested cell cycle in G2/M, accumulated polyploid cells that underwent mitotic catastrophe and induced p21-driven senescent-associated cell death. In U251 cells axitinib treatment stably arrested cell cycle in G2/M and increased polyploid cells that underwent mitotic catastrophe as above described, but the failure to induce p21 overexpression rescued glioma cells to axitinib-induced cell death. Furthermore, we demonstrated that overexpression of p21 in U251 cells restores the sensitivity of glioma cells to axitinib and induces a necrotic cell death as shown by the typical DNA smear and the lack of procaspase-3 activation. These data are in agreement with previous findings demonstrating that p21 is able to bind and inhibit the activity of proteins directly involved in the induction of apoptosis, including procaspase [43]. Further, the overexpression of the cell cycle inhibitor p21, at time of Chk1 activation shutdown, represented a prerequisite to drive glioma cells to death. Similarly, in PC-3 prostate cancer cells, retrovirus-mediated transduction of p21 induces cell death by suppressing Chk1 activation and DDR [42]. On the other hand, the silencing of p21 made T98 cells resistant to axitinib therapy confirming a pivotal role of p21 in the glioma cellular response to this TKI.

Bortezomib and the new proteasome inhibitor, marizomib, have been found to induce increased p21 levels in GBM [44]. The present study demonstrated that the advantageous schedule to treat glioma cells *in vitro* is the axitinib and bortezomib coadministration. We also characterized the molecular mechanisms involved in the synergistic effect between these drugs. We demonstrated that bortezomib when administered at nanomolecular, no toxic doses in combination with suboptimal dose of axitinib, promotes cytotoxic activity both in axitinib-sensitive and -resistant glioma cell lines by increasing p21 protein levels, as supported by the p21 silencing experiments. The axitinib plus bortezomib synergistic effect was associated also with Ser345-Chk1 activation and H2AX phosphorylation mainly in U87 and U251 cell lines, although with different kinetics. This combined treatment induced mitotic catastrophe and ROS-mediated DNA-damage senescence-associated necrosis in U87 and T98 glioma cells. Interestingly in axitinib-resistant U251 cells, the administration of bortezomib made the cells sensitive to the TKI, by increasing Ser345-Chk1, H2AX phosphorylation and triggering p21 overexpression, with the induction of ROS-dependent necrosis. These results suggest that different levels of ROS and sensitivity of cells to drug-induced oxidative stress, may influence cell death modalities [32, 45]. Overall, these results demonstrated that H2AX, Ser345-Chk1 and p21 phosphorylation levels, as well as ROS generation induced by axitinib alone or in combination with bortezomib, promoted the sensitivity of glioma cells, and overcame the resistance, through the activation of different cell death outcomes.

Finally, although promising results were recently obtained by Duerinck *et al*. (increased 6-month PFS rate and median OS) in a randomized phase II trial [7], a long-term disease control or cures have not been still obtained in glioma patients. On the other hand, bortezomib has been recently demonstrated to sensitize glioma cells to apoptotic cell death *in vitro* [21, 22]. However, also for the ability of high doses of bortezomib to stimulate the angiogenesis of glioma stem-like cells [22], the *in vivo* bortezomib-induced effects are conflicting. The efficacy of bortezomib in combination with chemotherapeutic drugs (e.g., tamoxifen, temozolomide) or radiotherapy is limited [46]. Thus, the need to improve the efficacy of treatment by constructing a rationally directed combination strategy to achieve meaningful anti-glioma activity represents a key point.

Although the extrapolation of *in vitro* data to the clinical setting should be considered with caution, our results obtained administering suboptimal dose of bortezomib demonstrated to not affect cell viability nor VEGFA expression, combined with low doses of the anti-angiogenetic TKI, axitinib may provide a rationale for the ongoing clinical investigation. Moreover, this combinatorial approach could be able to overcome the above-mentioned limitations by enhancing the cytotoxicity against glioblastoma.

## MATERIALS AND METHODS

### Cell line culture

The p53-wild type U87MG glioma cell line was obtained from American Type Culture Collection (LGC Promochem, Teddington, UK). The human p53-mutant T98 and U251 glioma cell lines were obtained from Cell Bank Interlab Cell Line Collection (ICLC, Italy). Glioma cells were grown in Eagle's Minimum Essential Medium (Lonza Bioresearch, Basel, Switzerland) supplemented with 10% (v/v) heat-inactivated fetal bovine serum (FBS), 2mM L-glutamine and 100 IU/ml of penicillin, 100 μg of streptomycin (Lonza), 1 mM sodium pyruvate (Lonza) and non-essential amino acids (Lonza). Cell lines were maintained at 37°C, 5% CO_2_ and 95% of humidity.

### Reagents

Axitinib (Inlyta®) was kindly provided by Pfizer (New York, NY). Bortezomib (BORT) was provided by Janssen-Cilag International N.V. (Beerse, Belgium). Mouse monoclonal anti-glyceraldehyde-3-phosphate dehydrogenase (GAPDH) Ab was from Origene (Rockville, MD). Mouse anti-p21 antibody (Ab) was purchased from Santa Cruz Biotechnology (Santa Cruz, CA). Rabbit anti-phospho-histone H2AX (Ser139), anti-Chk1-Ser345, anti-Chk1 and anti-caspase-3 Abs were purchased from Cell Signaling Technology (Danvers, MA). The following secondary antibodies were used: horseradish peroxidase (HRP)-conjugated anti-mouse IgG and HRP-conjugated anti-rabbit IgG (Cell Signaling Technology). Annexin V-fluorescein isothiocyanate (Annexin V-FITC) was purchased from eBioscience (Hatfield, UK). 5-dodecanoylaminofluorescein di-β-D- galactopyranoside (C12FDG) were from Invitrogen (San Diego, CA, USA). Bafilomycin A1, dimethyl sulfoxide (DMSO, used as vehicle), Hoechst 33258, propidium iodide (PI), ribonuclease A, 5-bromo-4-chloro-3-indolyl β-D- galactopyranoside (X-Gal), N-acetyl-L-cysteine (NAC), 10-N-nonyl acridine orange (NAO) and 3-(4,5-dimethylthiazol-2-yl)-2,5-diphenyltetrazolium bromide (MTT) were from Sigma Aldrich (St. Louis, MO). 5,5,6,6-tetrachloro-1,1,3,3-tetraethyl benzimidazolylcarbocyanine iodide (JC-1) was from Invitrogen (Carlsbad, CA).

### MTT assay

The colorimetric MTT assay was used to evaluate cell viability. Three ×10^4^ cells/ml were seeded into 96-well plates. After 1 day of incubation, compounds or vehicles were added. To evaluate the single-agent treatment, the cells were exposed to axitinib or bortezomib alone for up to 72 h, and the half maximal inhibitory concentration (IC_50_) was considered as the concentration resulting in 50% cell growth inhibition compared with the untreated control cells. To evaluate the cytotoxic effects of the combined treatment, the cells were treated with two different schedules: pretreated with axitinib for 72 h, washed, replated and treated with bortezomib for 72 h; treated concomitantly with axitinib and bortezomib for up to 72 h. In some experiments cells that were treated with axitinib and/or bortezomib for 72 h, were incubated in drug-free medium for 72 h. Four replicates were used for each treatment. At the indicated time point, cell viability was assessed by adding 0.8 mg/ml of MTT to the media. After 3 h supernatants were discarded and coloured formazan crystals dissolved with 100 μl/well of DMSO, were read by an enzyme-linked immunosorbent assay reader (BioTek Instruments, Winooski, USA). Four replicates were used for each treatment. Vehicle data were omitted since no effects were observed as respect to untreated cells.

Synergistic activity of the axitinib-bortezomib combination was determined by the isobologram and combination index (CI) methods (CompuSyn Software, ComboSyn, Inc. Paramus, NJ 2007). The CI was used to express synergism (CI < 1), additivity (CI = 1) or antagonism (CI > 1) and was calculated according to the standard isobologram equation [47].

### Cell cycle analysis

Three x10^4^ glioma cells/ml were treated with the appropriate drugs, collected and fixed in 70% ethanol and then washed with staining buffer (phosphate-buffered saline, PBS, 2% FBS and 0.01% NaN_3_). Next, the cells were treated with 100 μg/ml ribonuclease A solution, incubated for 30 min at 37°C, stained for 30 min at room temperature with PI 20 μg/ml and then analysed by flow cytometry using linear amplification.

### Western blot analysis

Cells were lysed in lysis buffer containing protease inhibitor cocktail (Sigma Aldrich). Lysates were separated on 8-14% SDS polyacrylamide gel and transferred onto Hybond-C extra membranes (GE Healthcare). Membrane were incubated overnight at 4°C in primary Abs (anti-phospho-H2AX 1:1000, anti-Chk1-Ser345 1:1000, anti-Chk1 1:1000, anti-p21 1:300, anti-caspase-3 1:1000, anti-GAPDH 1:8000), followed by the incubation (room temperature, 1 h) with HRP-conjugated anti-rabbit or anti-mouse secondary Abs. Peroxidase activity was visualized with the LiteAblot ®PLUS and LiteAblot® TURBO (EuroClone, Milan, Italy) kits and densitometric analysis was carried out by a Chemidoc using the Quantity One software (Bio-Rad, Hercules, CA).

### Senescence analysis

We performed the senescence analysis by both microscope and flow cytometry to evaluate the senescence-associated β-galactosidase activity. Treated cells were fixed for 5 min at room temperature in 3% formaldehyde and incubated overnight at 37°C without CO2 with fresh SA-β-Gal stain solution: 1 mg/mL X-Gal, 150 mM NaCl, 2 mM MgCl2, 40 mM citric acid, 5 mM sodium phosphate (pH 6.0), 5 mM potassium ferrocyanide, and 5 mM potassium ferricyanide. Senescent cells were identified as blue-stained cells by standard light microscopy. Photographs were acquired and analyzed by an Olympus BX51 microscope (Hamburg, Germany) using magnification 40x.

Relatively to flow cytometry, we performed the assay using the fluorogenic substrate C_12_FDG. Drug-treated cells were incubated for 1 h at 37°C and 5% CO_2_ with 100 nM bafilomycin A1 in culture medium to induce lysosomal alkalinization at pH 6 and, then, for 1 h with 33 μM C12FDG. Samples were immediately analyzed using FACScan cytofluorimeter using the CellQuest software. The C_12_-fluorescein signal was measured on the FL-1 detector, and β-galactosidase activity was estimated as percentage of positive cells. Thereafter, in some experiments, cells were also incubated with 20 μg/ml PI followed by biparametric FACS analysis using the CellQuest software.

### Mitochondria staining

To determine mitochondrial mass mitochondrial staining was performed by 5,50,6,60-tetrachloro-1,10, 3,30-tetraehylbenzimidazolylcarbocyanineiodide (JC-1) staining [27]. Briefly, treated cells were incubated for 10 min at room temperature with 10 μg/ml of JC-1. Samples were analysed using a FACScan cytofluorimeter with CellQuest software.

To further support the JC1 assay, we used acridine orange 10-nonyl bromide (NAO), a metachromic dye that fluoresces at 533 nm [48]. Treated cells were incubated at 37°C for 30 min with 0.1 µM of NAO, washed and analyzed by flow cytometry.

### Annexin V and PI staining

Cell death was evaluated using Annexin V-FITC and PI staining followed by flow cytometry and FACS analysis. Three x10^4^ cells/ml were treated with vehicle, axitinib and/or bortezomib for up to 72 h. After treatment, cells were stained with 5 μl of Annexin V-FITC and 20 μg/ml PI for 10 min at room temperature and washed once with binding buffer (10 mM Hepes/NaOH pH 7.4, 140 mM NaCl, 2.5 mM CaCl2). The percentage of positive cells determined over 10,000 events was analyzed on a FACScan cytofluorimeter using the CellQuest software.

### DNA fragmentation assay

Glioma cells were treated as above described for up to 72 h, and genomic DNA was extracted using the Apoptotic DNA Ladder detection Kit (Life Technologies Italia, Monza, Italia). The purified samples were then subjected to electrophoresis on a 1.25% agarose gel, and DNA was stained with ethidium bromide. Ultraviolet spectroscopy at 302 nm was used to obtain the results.

### ROS production

Cells were cultured for up to 72 h with axitinib or vehicle. In some experiments, cells were preincubated for 3 h with 10 mM NAC. Cells were then washed with PBS, pulsed with DCFDA for 10 min at 37°C, 5% CO_2_, and analyzed by FACScan cytofluorimeter using the CellQuest software.

### Immunofluorescence and microscopic analysis

Cells cultured for up to 72 h were washed with PBS and fixed in 4% formaldehyde for 10 min at room temperature. For nuclei analysis, cells were stained with Hoechst 33258 and examined by using the Olympus BX51 microscope. For morphological analysis, cells were stained with haematoxylin for 1 min and washed again before staining with eosin for 30 seconds. Slides were mounted with 50% glycerol, sealed and observed under Olympus BX51microscope.

### Cell transfection

U251 cells were plated at a density of 2×10^5^ cells/ml. After overnight incubation, transfections were achieved with 7.5 μl/ml of the reagent TransIT-X2 (Mirus MIR-6003, OriGene, Rockville, MD) and 2.5 μg/ml of pCMV-p21 or pCMV empty (pCMV) vectors according to the manufacturer's instructions. The cells were harvested at 72 h post-transfection for analysis. The efficiency of transfection was evaluated by western blot analysis.

### p21 silencing

Small interfering RNAs (siRNAs) targeted to p21 (siCDKN1A) and a non-silencing siRNA (NC1) served as control were purchased from Integrated DNA Technologies (Leuven, Belgium). T98 cells were plated at a density of 2×10^5^ cells/ml. After overnight incubation, transfections were achieved with 7.5 μl/ml of the reagent TransIT-X2 and 10 nM of siCDKN1A or NC1 (negative control) according to the manufacturer's instructions. The cells were harvested at 72 h post-transfection for analysis. The efficiency of transfection was evaluated by western blot analysis.

### RT-PCR analysis

Total RNA was extracted with the RNeasy Mini Kit (Qiagen), and cDNA was synthesized using the High- Capacity cDNA Archive Kit (Applied Biosystems, Foster City, PA) according to the manufacturer's instructions. Quantitative real-time polymerase chain reaction (qRT- PCR) for VEGFA was performed using the iQ5 Multicolor Real-Time PCR Detection System (Bio-Rad, Hercules, CA). PCR reaction was performed with RT2SYBRGreen qPCT mastermix (Qiagen) using 1 μl of cDNA for reaction, following the amplification protocol described in the manufacture's instruction. RT2 qPCR Primer assays (Qiagen) were used for target gene amplification. All samples were assayed in triplicates in the same plate. Measurement of GAPDH levels was used to normalize mRNA contents, and target gene levels were calculated by the 2^-ΔΔCt^ method.

### Statistical analysis

The statistical significance was determined by Student's t-test and by one way ANOVA. The statistical analysis of IC_50_ levels was performed using Prism 5.0a (Graph Pad).

## SUPPLEMENTARY MATERIALS FIGURES


