# Supplementary material for: Axitinib induces senescence-associated cell death and necrosis in glioma cell lines: The proteasome inhibitor, bortezomib, potentiates axitinib-induced cytotoxicity in a p21(Waf/Cip1) dependent manner
Source: Oncotarget. 2016 Dec 1;8(2):3380–95. doi: 10.18632/oncotarget.13769 (PMC5356889; doi:10.18632/oncotarget.13769)
Supplement: Supplementary file 1 [file oncotarget-08-3380-s001.pdf]

Axitinib induces senescence-associated cell death and necrosis in glioma cell lines: The proteasome inhibitor, bortezomib, potentiates axitinib-induced cytotoxicity in a p21(Waf/Cip1) dependent manner

SUPPLEMENTARY FIGURES

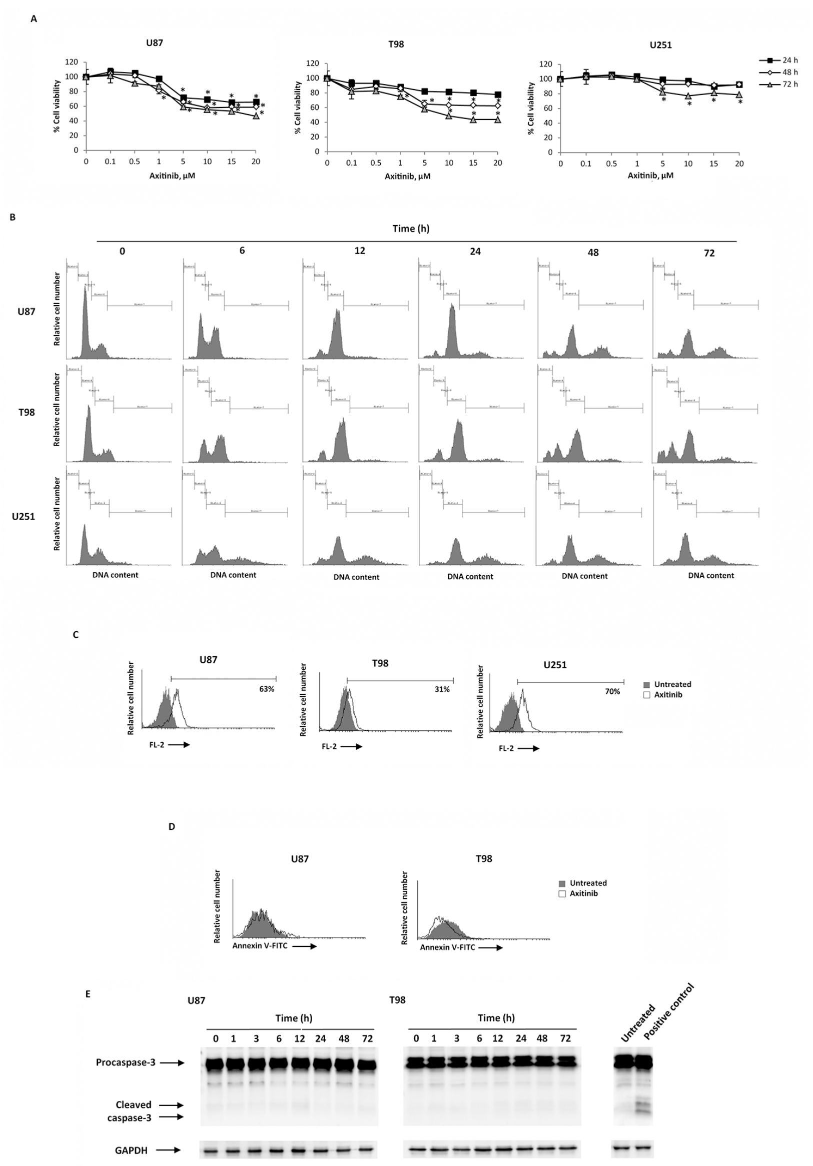

Supplementary Figure S1: Axitinib effects on glioma cell lines. A. U87, T98 and U251 cell lines were cultured up to 72 h with different doses of axitinib. Cell viability was determined by MTT assay. Data shown are expressed as mean ± SD of three separate experiments; *$p<0.01$ *vs* untreated cells. **B.** Representative cell cycle distribution in glioma cells treated with axitinib (5 μM for U87 and T98, 15 μM for U251) for up to 72 h. **C.** Cells were treated with axitinib for 72 h and then stained with NAO, used to evaluate total mitochondrial mass. NAO staining was quantified using flow cytometry and is displayed as a histogram. Data are expressed as percentage of NAO positive cells with respect to untreated cells. **D.** U87 and T98 cells were treated as above described and then Annexin V staining was analyzed by flow cytometry. Histograms are representative of one of three separate experiments. **E.** Representative immunoblot of caspase-3 in glioma cells treated as above described. GAPDH was used as loading control.

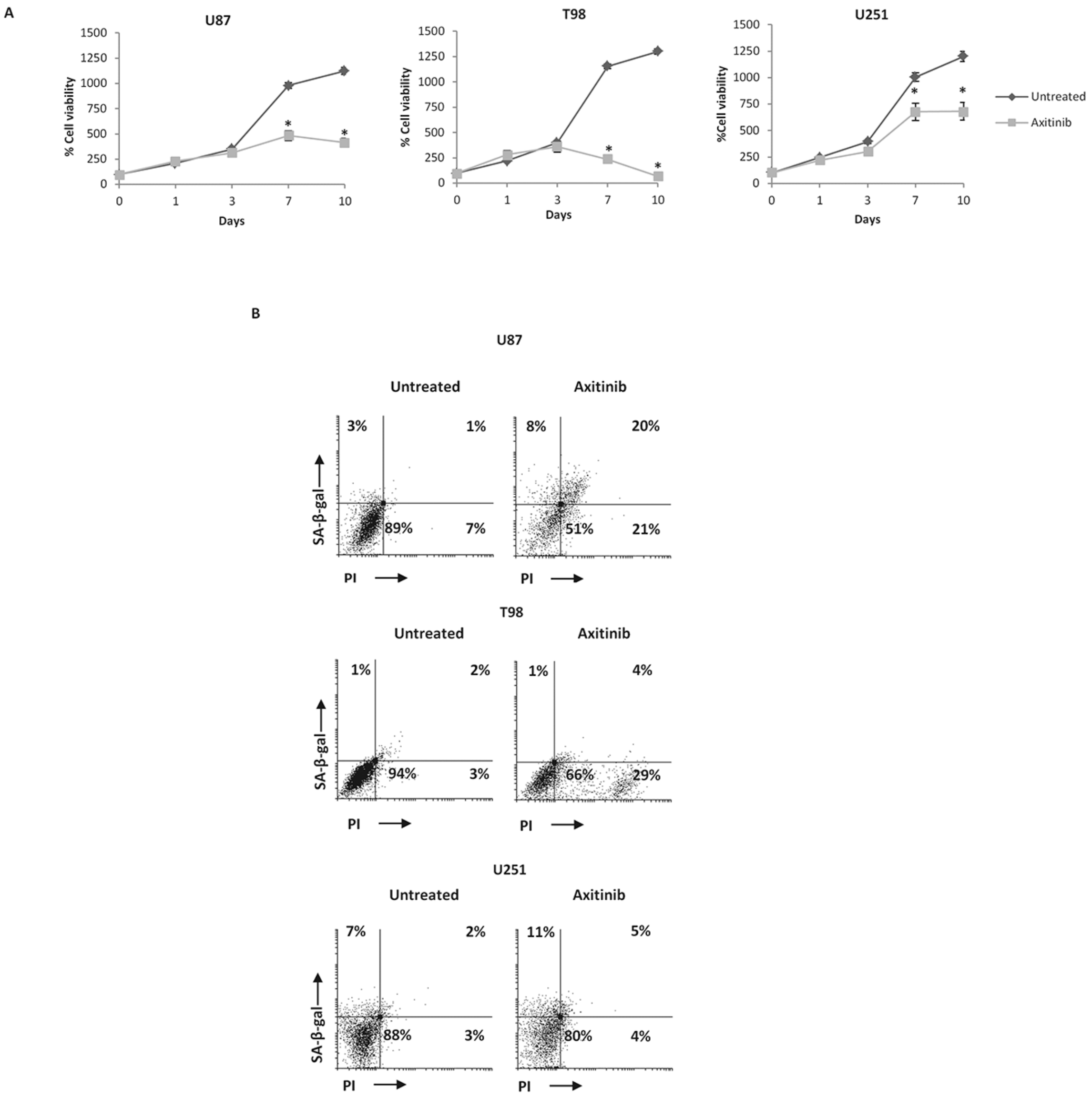

Supplementary Figure S2: Axitinib induces an irreversible effects and glioma cell death. A. U87, T98 and U251 cells were treated with axitinib (5 µM for U87 and T98, 15 µM for U251) for 72 h, then washed to remove the drug, replated and incubated for additional 10 days in fresh media. Cell viability was determined by MTT assay. Data shown are expressed as mean ± SD of three separate experiments; *$p<0.01$ *vs* untreated cells. **B.** Flow cytometric analysis on glioma cells cultured as above described was performed by C_{12}FDG and PI double-staining. Data represent the percentage of PI and/or SA-β-galactosidase positive cells and are representative of one of three separate experiments.

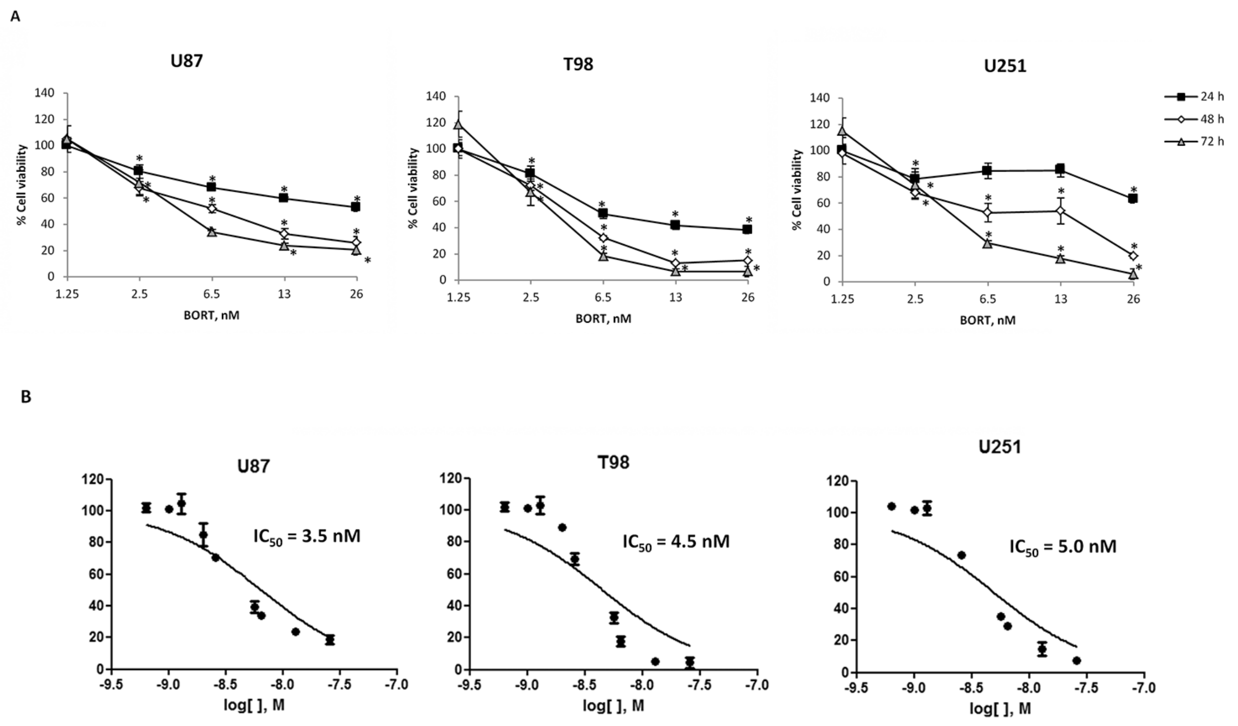

Supplementary Figure S3: Bortezomib inhibits glioma cell lines viability. A. U87, T98 and U251 cell lines were cultured up to 72 h with different doses of bortezomib. Cell viability was determined by MTT assay. Data shown are expressed as mean ± SD of three separate experiments; *p<0.01 *vs* untreated cells. **B.** U87, T98 and U251 glioma cell lines were cultured for 72 h with different doses of bortezomib. Cell viability was determined by MTT assay. Data shown are expressed as mean ± SE of three separate experiments.

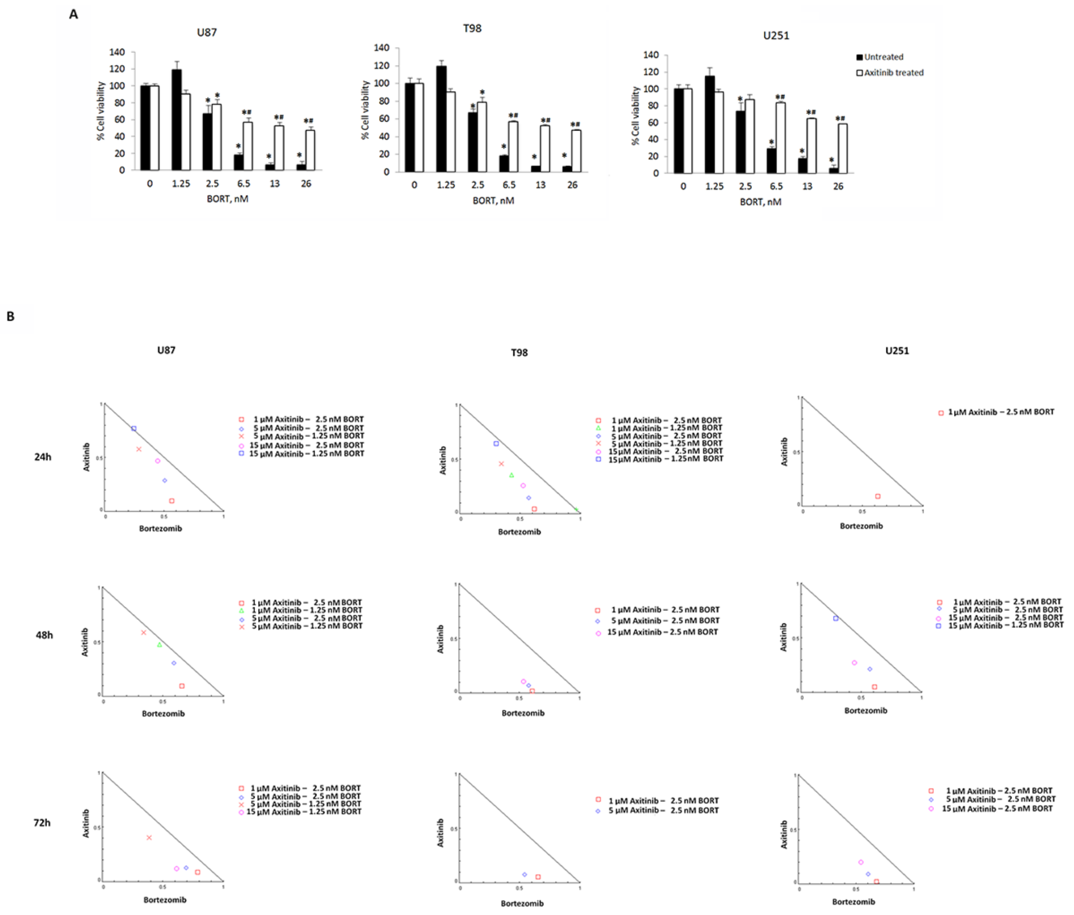

Supplementary Figure S4: Differential schedule administration of axitinib and bortezomib. A. U87, T98 and U251 cell lines cultured for 72 h with axitinib (5 µM for U87 and T98, 15 µM for U251) were washed, replated and treated for 72 h with different doses of bortezomib. Cell viability was determined by MTT assay. Data shown are expressed as mean ± SD of three separate experiments; *p<0.01 *vs* untreated cells; #p<0.01 *vs* axitinib pretreated cells. **B.** Isobologram analysis assessing the effects of simultaneous combinations of axitinib with bortezomib. U87, T98 and U251 glioma cell lines were cultured up to 72 h with 1, 5 and 15 µM axitinib and/or 1.25, 2.5 and 6.5 nM bortezomib. Cell viability was determined by MTT assay.

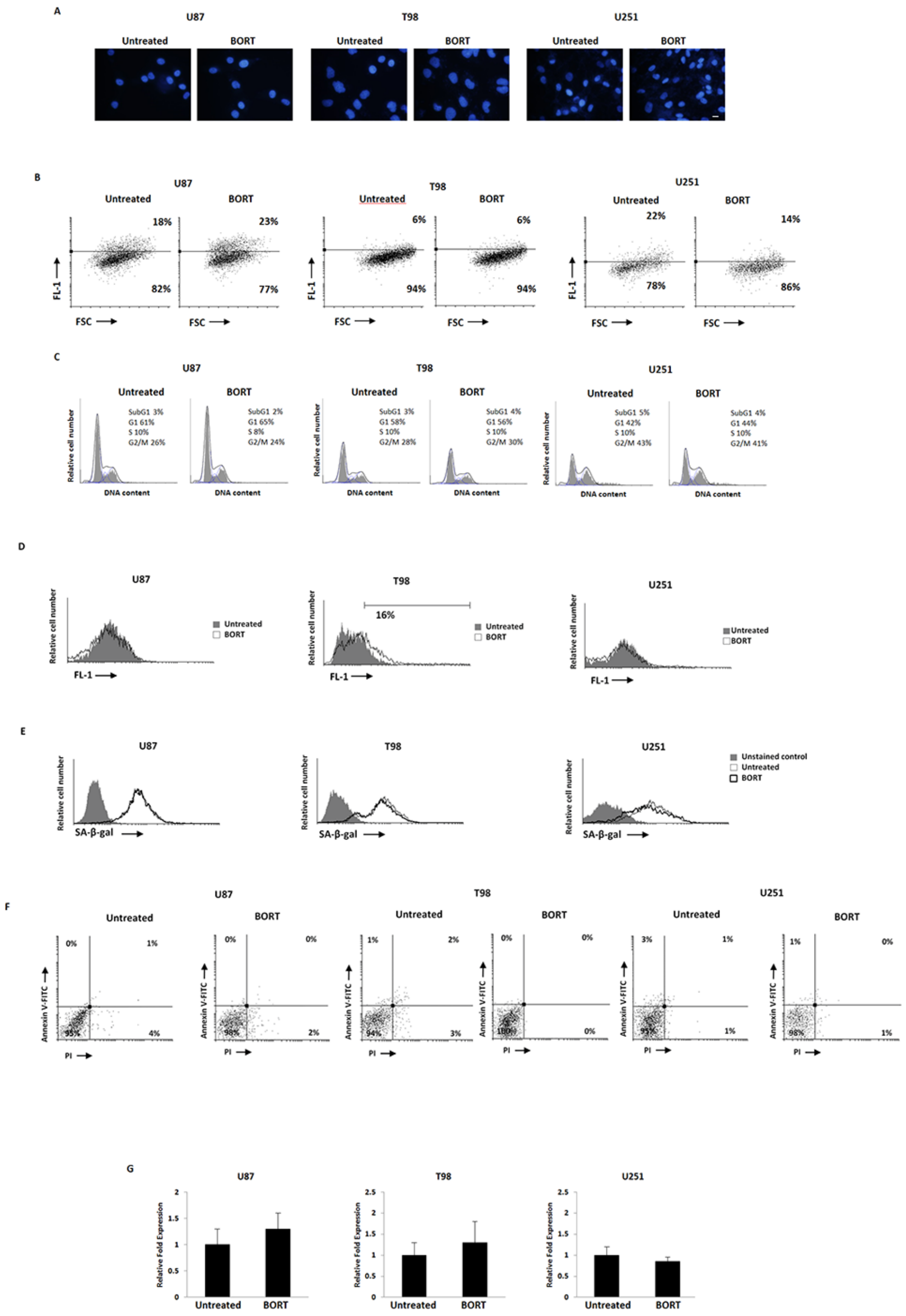

Supplementary Figure S5: Effects of 2.5 nM bortezomib on glioma cell lines. **A.** Nuclei of glioma cells treated with 2.5 nM bortezomib for 72 h were stained with Hoechst 33258 and then analyzed on ten random fields. Cells were observed under a fluorescence microscope. Bar: 50 µM. **B.** Change in JC1 green (FL-1) respect to FSC parameter in cells treated with bortezomib as above described was detected by flow cytometer. **C.** Representative cell cycle phases in cells treated as above described. **D.** ROS generation in glioma cell lines at 72 h after bortezomib treatment. Cells were stained with DCFDA before flow cytometric analysis. Data are expressed as percentage of DCFDA positive cells with respect to untreated cells. **E.** Glioma cells were treated with bortezomib for 72 h. Flow cytometric analysis was performed by C_{12}FDG staining. Histograms are representative of one of three separate experiments. **F.** Biparametric flow cytometric analysis were performed on glioma cell lines treated for 72 h with bortezomib, by Annexin V-FITC and PI staining. **G.** The VEGFA mRNA levels were determined by qRT-PCR on glioma cells treated with axitinib for 72 h. GAPDH was used for normalization. Data are expressed as relative fold with respect to untreated cells used as control. Data are expressed as mean ± SD.

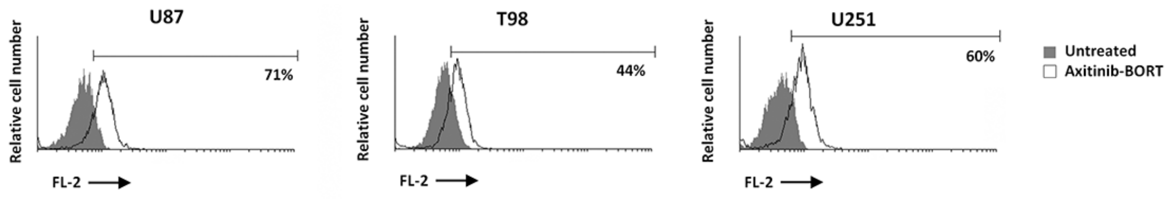

Supplementary Figure S6: The combination axitinib-bortezomib induces an increased mitochondrial mass. Cells were treated with axitinib (5 µM for U87 and T98, 15 µM for U251) and bortezomib (2.5 nM) combination for 72 h and then stained with NAO. Data are expressed as percentage of NAO positive cells with respect to untreated cells.

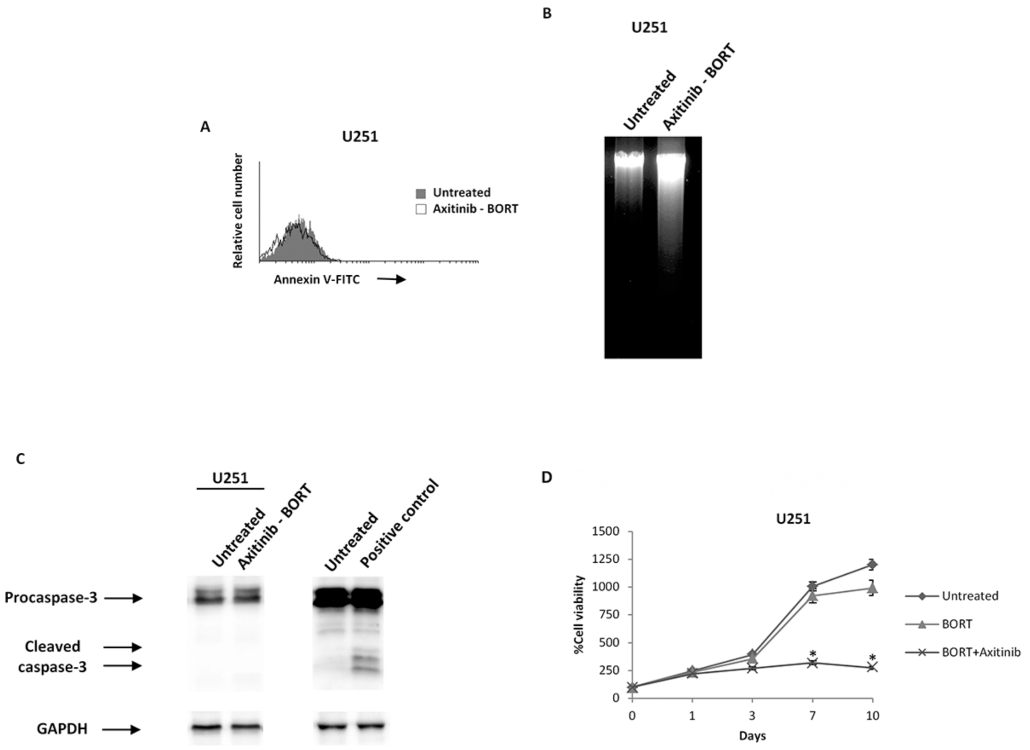

Supplementary Figure S7: Combined treatment with axitinib-bortezomib induces necrotic cell death in U251 cell lines. **A.** Flow cytometic analysis of Annexin V-FITC staining in U251 cells treated with the combination axitinib (15 µM) - bortezomib (2.5 nM). **B.** Representative agarose gel electrophoresis of DNA extracts obtained from U251 glioma cells after 72 h of treatment for assessment of DNA fragmentation **C.** Western blot analysis of caspase-3 protein levels in U251 glioma cells cultured for 72 h as above described. Blots are representative of one of three separate experiments. GAPDH was used as loading control. **D.** U251 cells were treated with axitinib (15 µM) alone or in combination with bortezomib (2.5 nM) for 72 h, then washed to remove the drug, replated and incubated for additional 10 days in fresh media. Cell viability was determined by MTT assay. Data shown are expressed as mean ± SD of three separate experiments; *p<0.01 *vs* untreated cells.

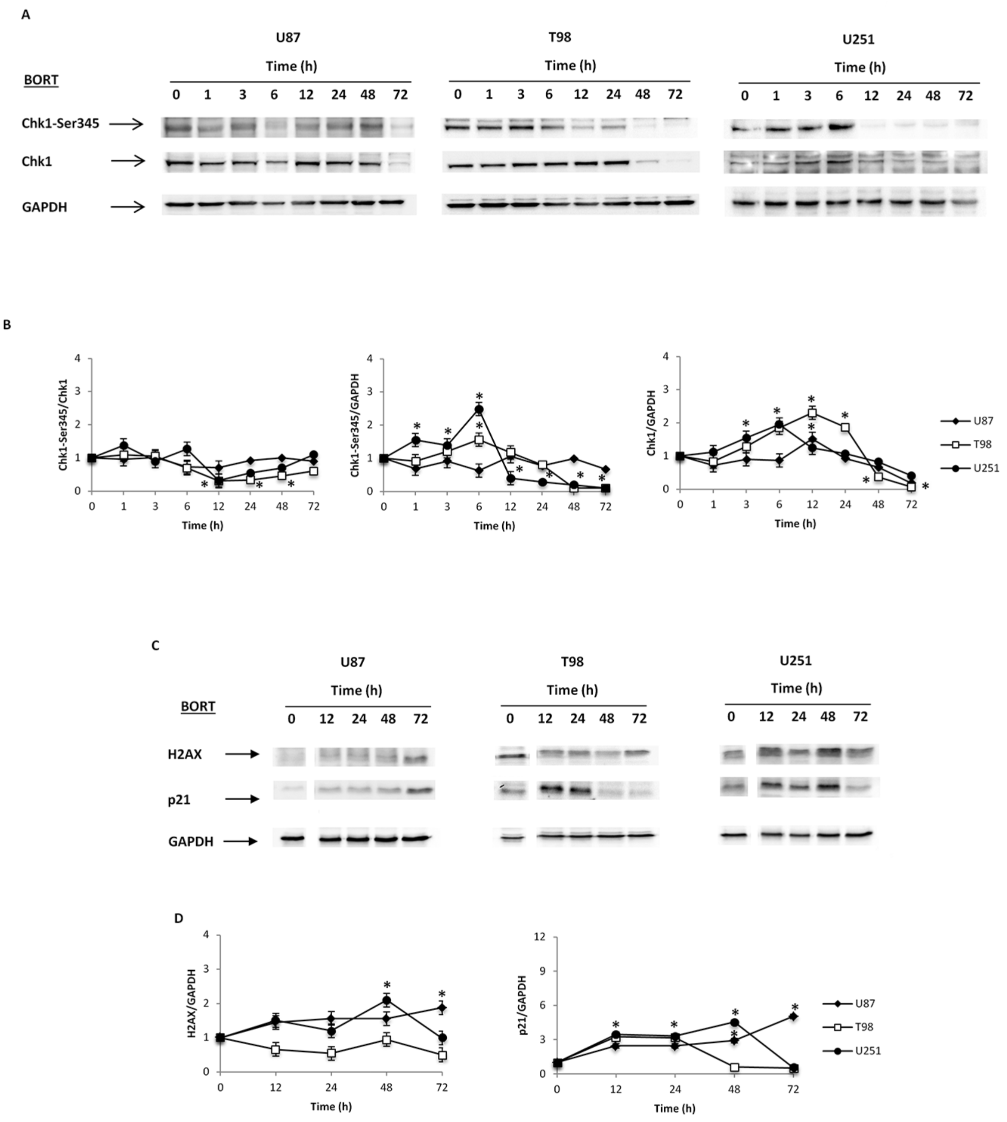

Supplementary Figure S8: Bortezomib induces DNA damage and p21 protein increase. A. Western blot analysis of Chk1-Ser345 and Chk1 protein levels in glioma cells after treatment with bortezomib 2.5 nM. Blots are representative of one of three separate experiments. **B.** Chk1-Ser345 and Chk1 densitometry values were normalized to GAPDH used as loading control. The Chk1-Ser345 protein levels were also determined with respect to Chk1 levels. Densitometric values shown are the mean ± SD of three separate experiments. *$p<0.01$ *vs* untreated cells. **C.** Western blot analysis of H2AX and p21 protein levels in glioma cells after bortezomib treatment. Blots are representative of one of three separate experiments. **D.** Quantitative representation of the experiment reported in panel C. H2AX and p21 densitometry values were normalized to GAPDH used as loading control. Densitometric values shown are the mean ± SD of three separate experiments. *$p<0.01$ *vs* untreated cells.
